# Tastant-receptor interactions: insights from the fruit fly

**DOI:** 10.3389/fnut.2024.1394697

**Published:** 2024-04-11

**Authors:** Christian Arntsen, Jacqueline Guillemin, Kayla Audette, Molly Stanley

**Affiliations:** Department of Biology, University of Vermont, Burlington, VT, United States

**Keywords:** taste, gustation, chemosensation, *Drosophila melanogaster*, taste receptor, gustatory receptor, tastant

## Abstract

Across species, taste provides important chemical information about potential food sources and the surrounding environment. As details about the chemicals and receptors responsible for gustation are discovered, a complex view of the taste system is emerging with significant contributions from research using the fruit fly, *Drosophila melanogaster*, as a model organism. In this brief review, we summarize recent advances in *Drosophila* gustation and their relevance to taste research more broadly. Our goal is to highlight the molecular mechanisms underlying the first step of gustatory circuits: ligand-receptor interactions in primary taste cells. After an introduction to the *Drosophila* taste system and how it encodes the canonical taste modalities sweet, bitter, and salty, we describe recent insights into the complex nature of carboxylic acid and amino acid detection in the context of sour and umami taste, respectively. Our analysis extends to non-canonical taste modalities including metals, fatty acids, and bacterial components, and highlights unexpected receptors and signaling pathways that have recently been identified in *Drosophila* taste cells. Comparing the intricate molecular and cellular underpinnings of how ligands are detected *in vivo* in fruit flies reveals both specific and promiscuous receptor selectivity for taste encoding. Throughout this review, we compare and contextualize these *Drosophila* findings with mammalian research to not only emphasize the conservation of these chemosensory systems, but to demonstrate the power of this model organism in elucidating the neurobiology of taste and feeding.

## Introduction

The chemical sense of taste allows animals to evaluate their food options to encourage the consumption of beneficial nutrients and avoidance of potential toxins. Since gustation links the environment to nutrition and fitness, it is not surprising that this sense is well-conserved across a wide range of animals, from humans to fruit flies (1). The concept that certain chemicals elicit distinct taste perceptions can be traced back to the earliest philosophers, but a clear understanding of the molecular and cellular basis of taste only started to emerge in the early 2000s. Over the last two decades, there has been extensive research into identifying the receptors responsible for the “five basic tastes”: sweet, bitter, salty, sour, and umami (2). Many details of these canonical taste modalities are well-established in both mammalian and non-mammalian model organisms, including the fruit fly, *Drosophila melanogaster* (1, 3–5). *Drosophila* is a powerful model organism in neurobiology research that has continued to advance our understanding of gustation due to the ability to record taste cell activity *in vivo* from a single neuron or a complete set of specific taste cells (6–8). Readily available genetic tools also allow for investigation into the role of taste receptors in cellular physiology and chemosensory behaviors (9–11). This review introduces the *Drosophila* taste system and describes recent insights into novel tastant-receptor interactions for both canonical and non-canonical taste modalities with comparisons to mammalian gustation.

## The fruit fly taste system

In both mammals and *Drosophila*, primary chemosensory cells initiate taste sensation by evaluating a food source’s chemical properties. The mammalian gustatory system uses taste receptor cells (TRCs), modified epithelial cells found in taste buds throughout the oral cavity. TRCs detect chemicals and relay this information to afferent gustatory nerves (1), but the *Drosophila* peripheral nervous system directly detects tastants via gustatory receptor neurons (GRNs) (12). GRNs are distributed throughout the fly body, but the highest concentration of taste cells involved in feeding is located in the labellum, the *Drosophila* tongue homolog (5). Labellar GRNs express taste receptors that allow for the rapid identification of chemicals, promoting selectivity for compounds that represent specific taste modalities (13, 14), akin to lingual taste cells in mammals.

**Figure 1:**
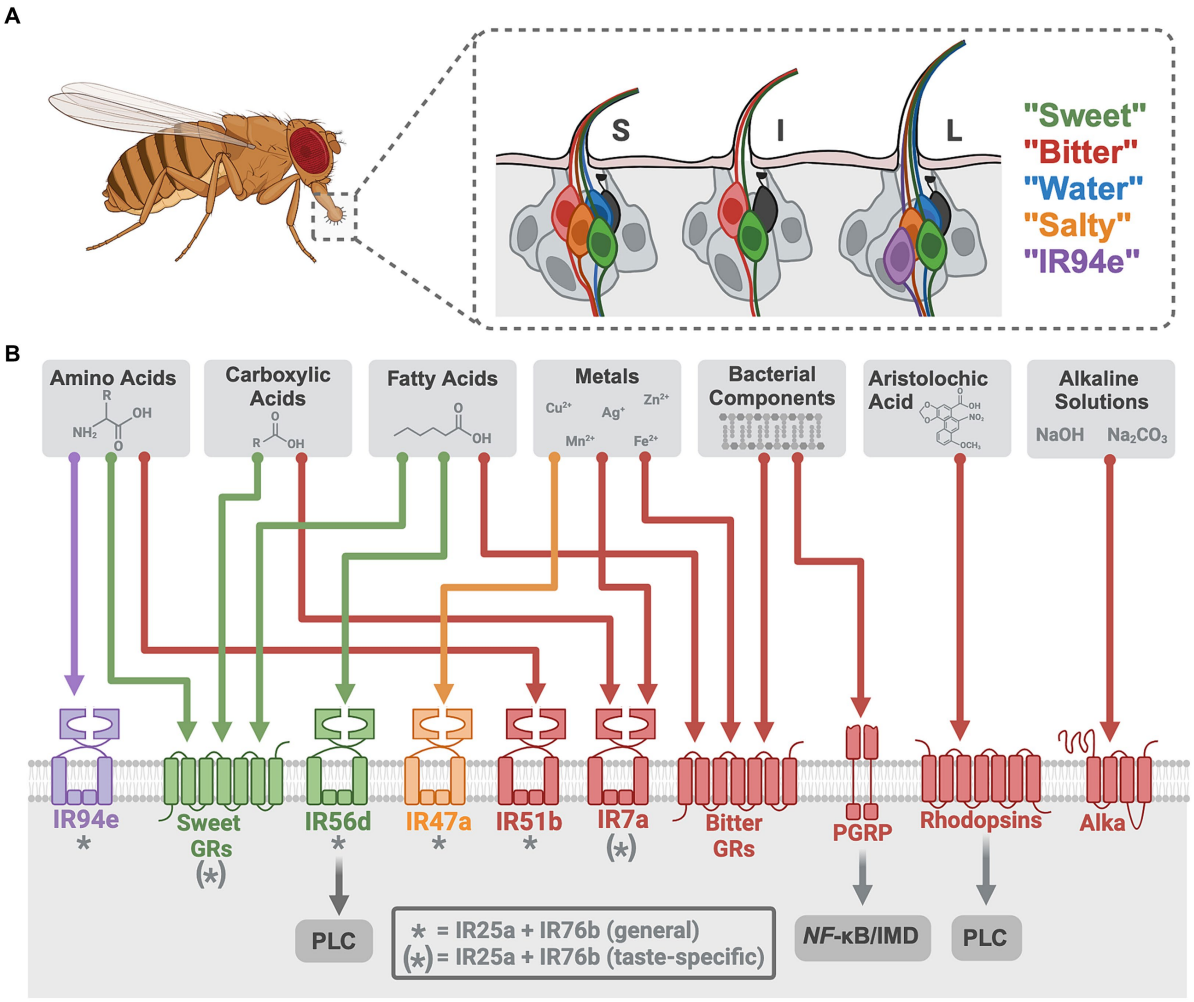
Neural encoding of novel tastant-receptor interactions in *Drosophila*. **(A)** Fruit flies will explore their chemosensory environment by extending their proboscis and interacting with tastants via sensillum on the labellum. (A inset) Three types of sensilla (S = short, I = intermediate, L = long) each contain two or four gustatory receptor neurons (GRNs) from five different cell types (color depicts each class, grouped by modality and receptor expression). Mechanosensory neurons are depicted in black. **(B)** Each GRN has a diverse array of chemosensory receptors (colored to match the GRN that houses it). Recently investigated tastants are depicted (gray boxes) with their specific receptors (colored arrows). * indicates “tuning” IRs that generally work with the ionotropic co-receptors. (*) indicates receptors that work alongside ionotropic co-receptors for only some of the taste modalities depicted. Tastant-receptor pairings that require intracellular pathways are depicted (gray arrows). Created with Biorender.com.

GRNs in the fruit fly labellum have been categorized into five groups based on their distinct receptor profiles and taste modality responsiveness: “sweet,” “bitter,” “water,” “salty,” and “IR94e” (15). These five GRN classes can be consistently mapped on a fly’s labellum across ~62 gustatory sensilla that are classified by size, each containing two or four GRNs (10, 16, 17) (Figure 1A). GRN axons project to the sub-esophageal zone in the brain (17, 18), where arborizations of both GRNs and motor neurons generate local circuits for taste-induced behavioral responses (19). The *Drosophila* whole-brain connectome (20–23) allows neural circuits to be traced from tastant-receptor activation through behavioral output to enhance our understanding of how taste information is encoded and modulated (24–28).

Gustatory processing commonly starts with two main classes of taste receptors in *Drosophila*: gustatory receptors (GRs) and ionotropic receptors (IRs). GRs consist of seven transmembrane domains, an intracellular N-terminus, and an extracellular C-terminus (29–32). Earlier studies that disrupted G-protein subunits in GR-expressing cells found a reduction in taste responses (33, 34), but recent research elucidating the structure of two sugar GRs has determined that they form tetrameric ligand-gated cation channels with peripheral ligand binding sites and a single central pore (35). The other class of *Drosophila* taste receptors, IRs, share structural similarities with synaptic, glutamate-gated ion channels: 3 transmembrane domains and a 2-lobed extracellular binding domain (36–38). IRs form heteromeric receptor complexes comprised of both co-receptors and “tuning” receptors that function as ligand-gated ion channels (39). In contrast to IRs and GRs, mammalian T1Rs and T2Rs are G-protein coupled receptors (GPCRs) (40–42), yet the repertoire of tastants acting via these receptors and their impact on behavior is remarkably conserved (1).

### Sweet, bitter, and salty taste

Direct activation of *Drosophila* “sweet” GRNs leads to appetitive feeding behaviors whereas “bitter” GRN activation produces avoidance (15, 19, 43), consistent with mammalian studies on these canonical tastes (1, 44, 45). *Drosophila* sugar receptors formed from 9 GR genes (sugar GRs) (46–51) detect mono- and disaccharides along with artificial sweeteners and molecules perceived as sweet to humans (52–54). The remaining 30+ GR genes form receptors in “bitter” GRNs (bitter GRs), detecting a range of bitter compounds (e.g., caffeine, lobeline, denatonium, and quinine) (55–58). Recent work in *Drosophila* has identified two non-canonical bitter signaling pathways for the detection of specific ligands, using rhodopsins and a peptidoglycan recognition protein (PGRP), that open new avenues for taste transduction (Table 1; Figure 1B). Rhodopsin GPCRs are typically light-sensitive with an opsin protein and retinal chromophore, but three rhodopsins (Rh1, Rh4, and Rh7) were found to function as taste receptors that do not require light or retinal (59). These rhodopsins detect aristolochic acid and activate “bitter” GRNs at particularly low concentrations through a phospholipase C (PLC) signaling cascade that involves TRPA1 (59). Mouse taste buds express some *opsin* RNA (75), suggesting these channels may have a conserved role in chemosensation. The other non-canonical pathway involves PGRPs, pattern recognition receptors traditionally involved in the immune response to pathogens. TRPA1 and canonical bitter GRs (Gr33a, Gr66a) were previously implicated in the detection of bacterial components (76, 77), but the newly described PGRP (PGRP-LB) expressed in the labellum specifically detects bacterial peptidoglycans. Unexpectedly, this receptor uses nuclear factor-κB (*NF*-κB)/immune deficiency (IMD)-dependent signaling to activate “bitter” GRNs (60). An interest in the role of oral taste receptors in microbial detection has emerged in mammalian research (78, 79), and this recent work in fruit flies highlights an unexpected role for *NF*-κB/IMD signaling in taste cells that impacts feeding choices (60).

**Table 1:** Recently described tastants and their receptors in the *Drosophila* labellum.

Tastant (s)	Receptor(s)	Details	References
Aristolochic acid: non-canonical bitter	Rhodopsins (Rh1, Rh4, Rh7)	No light or retinal required. Requires intracellular signaling	(59)
Bacterial peptidoglycan: non-canonical bitter	Peptidoglycan recognition protein (PGRP-LB)	Requires nuclear factor-κB (*NF*-κB)/immune deficiency (IMD)-dependent signaling	(60)
Metal ions: Cu^2+^, Ag^+^, Cd^2+^, Ni^2+^, Mn^2+^, Fe^2-3+^, Zn^2+^, Co^2+^	IR25a*, IR76b*, IR7a°, IR47a°, Bitter GRs	IR and/or GR complexes required. Receptor depends on the specific ion	(61–63)
Carboxylic acids: acetic, lactic, glycolic, citric	IR25a*, IR76b*, IR7a, Sugar GRs	Receptor complex depends on concentration. Some ligand specificity. Unclear if or how receptors work with OtopLa	(64–67)
Amino acids: 20 proteinogenic	IR25a*, IR76b*, IR51b°, IR94e°, Sugar GRs	Receptor complex depends on concentration. Some ligand specificity	(68, 69)
Alkaline solutions: NaOH, Na_2_CO_3_	Alkaliphile (Alka)	Cl^−^ channel gated by high pH	(70)
Fatty acids: hexanoic acid and other MCFAs, SCFAs, LCFAs	IR25a*, IR76b*, IR56d°, Sugar GRs, Bitter GRs	Receptor complex depends on concentration. Requires intracellular signaling (at least MCFA). Mechanisms for MCFA different from others	(71–74)

Receptors with the most consistent evidence are listed. * indicates broadly expressed IR co-receptors, ° indicates narrowly expressed IR “tuning” receptors that pair with the co-receptors. IR = Ionotropic Receptor, GR = Gustatory Receptor.

Recent advances in salt taste have revealed a complex taste transduction system that allows for concentration-dependent salt feeding in both mammals and fruit flies (80). A set of “high salt” or “salty” GRNs in the *Drosophila* labellum are specifically activated by high concentrations of various salt ions (15, 81, 82). Salt also activates other GRNs (“sweet,” “bitter,” and “IR94e”) while inhibiting “water” GRNs, producing a combinatorial code that can lead to flexible behaviors (15). Salt taste research highlights the role of IRs that use the broadly expressed co-receptors, IR25a and IR76b, plus a narrowly expressed “tuning” IR to form functional receptors that detect specific salt ions (15, 82–84). Canonical salt taste centers around NaCl and occasionally other mono- or divalent ions (80), but recent research has shifted focus to identify the taste mechanisms for other ions.

### Metal taste

Metals, including divalent and trivalent salt ions, have complex taste profiles (85–87) that have garnered increasing attention due to their accumulation in soil, crops, and foods from human activities (88, 89). Recent studies established that the human bitter taste receptor TAS2R7 acts as a metal cation receptor for detecting zinc and copper (90), yet this can only be demonstrated *in vitro*. Fruit flies avoid consuming metals and *in vivo* quantifications of neuronal activity reveal that metal ions activate taste cells through multiple receptors (Table 1; Figure 1B).

In *Drosophila*, some metal ions require only bitter GRs (Cu^2+^, Ag^+^) or IRs (Mn^2+^, Ni^2+^, Cd^2+^) for detection, while others require both (Zn^2+^, Co^2+^). Interestingly, cellular responses to iron involve both receptor types or solely IRs depending if it is in the Fe^2+^ or Fe^3+^ form, respectively (61). Cadmium sensitivity requires an IR complex in two types of GRNS: co-receptors (IR25a, IR76b) plus IR7a in “bitter” GRNs and the same co-receptors plus IR47a in “salty” GRNs (63). A recent brief report found an additional “tuning” receptor, IR56b, to be necessary for zinc avoidance (62), however, this receptor complex detects NaCl in “sweet” GRNs for attraction (83), so this is an unexpected result. Overall, this research in flies provides clear evidence that a range of individual metal ions have specific taste detection mechanisms. As metal contamination continues to rise, understanding gustatory pathways for metal ligands will become increasingly important across animals with relevance to environmental health and food safety.

### Sour taste

pH is an important indicator of food quality and sour taste describes the gustatory detection of acids. Recently, the Otop1 proton channel was identified as the “sour receptor” in mammals (91, 92), and a homolog, *OtopLa*, is expressed in fruit fly GRNs in the labellum (93, 94). While the discovery of Otop channels was an important breakthrough for sour taste, different acids have distinct taste qualities even at the same pH, suggesting there is more to sour taste than pH alone (95, 96).

Like humans, fruit flies show dose-dependent attraction or aversion to certain carboxylic acids (67). Weak organic acids, such as acetic acid, may have the ability to cross the membrane of taste cells to impact transduction by altering intracellular pH, but through unknown mechanisms (97, 98). In *Drosophila*, attractive concentrations of specific organic acids—acetic, lactic, glycolic, and citric—require taste receptors for the activation of “sweet” GRNs (64–66). At least one broadly expressed co-receptor, IR25a, is involved, along with sugar GRs for detecting organic acids (64–66) (Table 1; Figure 1B). Even at the same pH, these acids differentially activate *Drosophila* taste cells *in vivo*, indicating diverse receptor binding and/or abilities to cross cell membranes. Attempts to distinguish between the detection of pH and anion species show that IRs are largely involved with anion detection, whereas the sugar GRs are responsive to the change in pH (66). Ascorbic acid (Vitamin C), a distinct but related acidic compound, was also found to activate “sweet” GRNs through similar mechanisms (65). High concentrations of carboxylic acids are aversive (99), and IR7a in “bitter” GRNs is specifically required for acetic acid avoidance, without the need for IR co-receptors (67). While the cooperative role of OtopLa channels along with these receptors remains unclear, these findings underscore the dual activation of taste cells by acids through both receptors and proton influx.

### Alkaline taste

Since pH influences food quality, the ability to detect both basic and acidic pH levels would be advantageous. Previous mammalian studies on basic pH sensation focused on somatosensation (100), but humans show alkaline sensitivity on the tip of the tongue (101) and a recent study in rats found that sodium carbonate (Na_2_CO_3_) solutions activate taste nerves significantly more than Na^+^ alone. However, alkaline taste has not been well described. A recent study in *Drosophila* established the existence of alkaline taste, and identified a novel receptor required for the detection of basic solutions (70) (Table 1; Figure 1B). Alkaliphile (Alka) is a Cl^−^ channel gated by high pH that is necessary for alkaline taste (70). The Alka receptor is expressed in a subset of the “bitter” GRNs (~21%), but it is currently unclear what other cell types may express this receptor. Regardless, this study in flies establishes a novel tastant-receptor interaction for alkaline taste that may be relevant to mammals. Interestingly, the Otop1 proton channel for sour taste was recently found to be a candidate alkaline receptor *in vitro* (102), indicating a need for future comparative studies on basic and acidic pH detection mechanisms.

### Umami (amino acid) taste

Protein feeding is coupled with the chemical detection of amino acids. Umami taste is a specific savory sensation, usually associated with monosodium L-glutamate (MSG), an amino acid often found in foods at higher concentrations (103–105). The mammalian GPCR complex consisting of T1R1 + T1R3 is referred to as the “umami receptor” (45) and has a high sensitivity to glutamate in humans (106). In most vertebrates, this receptor is broadly responsive to amino acid ligands and amino acids can also activate sugar taste receptors, bitter taste receptors, or act through metabotropic glutamate receptors in multiple cell types (107–114). This combinatorial coding likely occurs in response to individual amino acids in a dose-dependent manner. Through *in vitro* assays, mammalian bitter receptors display dose-dependent activation by amino acids, however, some inconclusive results are attributed to the possibility of endogenous amino acid receptors in the cell line used for these experiments (109). *In vivo* studies in *Drosophila* circumvent these concerns and allow for a deeper understanding of the combinatorial coding for amino acid taste (Table 1).

Fruit flies require and consume amino acids based on internal state, such as mating status or nutritional deficiency (115). The IR co-receptors (IR76b and IR25a) are necessary for detecting most of the proteinogenic amino acids at various concentrations (68, 69, 116), and the “tuning” receptors identified for amino acid sensation to date are IR51b and IR94e. IR51b is a bitter cell-specific receptor that detects high concentrations of arginine, valine, leucine, tryptophan, isoleucine, lysine, and proline (68). IR94e receptors are integral for the detection of glutamate in various forms, and this “tuning” receptor is expressed in a newly described set of taste cells that induce mild feeding aversion (27, 69). A thorough description of the combinatorial coding for low concentrations (25 mM) of arginine reveals that “sweet” GRNs are activated through both sugar GRs (Gr5a, Gr61a, and Gr64f) and IR co-receptors (68) (Figure 1B). The overlap between sugar-sensing and amino acid-sensing resembles a pattern found in mammals (117).

A feature of the mammalian “umami receptor” is enhancement by purine-5′-nucleotides (IMP and GMP) (111, 114, 118), but this feature is not known to occur in fruit flies. Additionally, while no metabotropic glutamate receptors have been identified in fruit fly amino acid taste, the IRs are ancestrally related to ionotropic glutamate receptors (36–38, 119), suggesting a conserved use of glutamate receptors in chemosensation (107, 113, 120). In *Drosophila*, another intriguing element is that an odorant binding protein (OBP19b) secreted from nearby cells can bind certain amino acids to impact their detection by taste cells (121), but it is unclear how conserved this mechanism may be. Despite some differences from the mammalian system, the *Drosophila* model offers a way to study dose-dependent encoding of individual or groups of amino acids to better understand this canonical yet complex taste modality.

### Fatty acid taste

Fatty acids are highly energetic essential nutrients that are attractive to both mammals and *Drosophila* (71, 122–124). Initially, fat palatability was thought to be driven by texture and olfaction (125), but more recent research has highlighted the importance of gustation (126–128). In mice, CD36 is a fatty acid transporter expressed in taste buds that contributes to fatty acid preferences (129), and two GPCRs (GPR40 and GPR120) appear to function as lingual fat receptors (130). Although *Drosophila* homologs have not been discovered, GRNs in the labellum do detect fatty acids (71). Similar to carboxylic and amino acids, the cellular and behavioral responses to fatty acids in flies depend on concentration.

At low concentrations (~0.1%), hexanoic acid elicits appetitive responses in *Drosophila*, while at high concentrations (~1–2%), it prompts aversion (74). Hexanoic acid attraction is driven by “sweet” GRNs, requiring both IR56d and Gr64d (72–74, 131, 132). Aversion to hexanoic acid is controlled by “bitter” GRNs via three bitter receptors: Gr32a, Gr33a, and Gr66a (74) (Table 1). Recent work has also demonstrated that the fly gustatory system can distinguish between different classes of fatty acids based on chain length (73). While all classes of fatty acids require the IR co-receptors (IR25a and IR76b) for detection, medium-chain fatty acid (MCFA) taste requires “sweet” GRNs and the IR56d receptor, whereas short-chain (SCFA) and long-chain (LCFA) fatty acid taste does not (73). These findings indicate that IR56d is selective for MCFAs, while the co-receptors may function more broadly. However, a recent study questioned the involvement of IR25a and IR76b in the labellar response to the MCFA hexanoic acid (74). The molecular and cellular underpinnings of SCFA/LCFA detection and fatty acid discrimination remain unclear, but these complexities reflect a nuanced fatty acid taste encoding system that is sensitive to both concentration and subtle variations in molecular structure.

MCFA taste also requires intracellular signaling, as flies with a mutant *norpA*, a *Drosophila* homolog for PLC, have disrupted MCFA detection (71) (Figure 1B). Whether or not PLC signaling is necessary for SCFA and LCFA sensation is unknown. Furthermore, one study showed that the sugar GR, Gr64e, is an essential component of MCFA signal transduction, unexpectedly serving as a downstream component in the PLC pathway within “sweet” GRNs (133). Notably, a recent investigation found that Gr64e mutation did not affect electrophysiological responses to the MCFA hexanoic acid (74). Despite this discrepancy, activation of a secondary receptor via PLC mimics the mammalian fatty acid signaling cascade. Mice lacking PLC or TRPM5, a downstream receptor in the PLC cascade, lose their taste preference for fatty acids (134). Collectively, these results imply that PLC-mediated intracellular mechanisms underpin fatty acid gustation in both *Drosophila* and mammals, despite mammalian research primarily focusing on LCFAs which remain attractive at higher concentrations (130). *Drosophila* fatty acid taste emphasizes the conserved nature of macronutrient taste encoding and may prove valuable for informing future fat perception research to uncover more about this non-canonical taste modality that has many health implications.

## Discussion

Recent advances in gustation research using *Drosophila melanogaster* as a model organism have revealed several unexpected ligand-receptor interactions within the taste system that play crucial roles in chemosensation and behavior. The discovery of two novel receptor signaling types in bitter cells, through non-canonical rhodopsin and immune signaling, has revealed unexpected transducers for contact chemical cues. Moreover, the fly gustatory system contains a markedly complex set of receptors to detect specific metals, which may become increasingly relevant in this Anthropocene Epoch. The identification of receptors for carboxylic acid anions suggests a mechanism for sour taste that extends beyond proton detection, while a novel receptor for alkaline solutions highlights the role of gustation in discerning a broader pH spectrum. The ability to study intact taste cells in awake flies has provided key insights into the concentration-dependent nature of ligand detection across multiple receptors and cell types for carboxylic, amino, and fatty acids that imply combinatorial taste coding mechanisms to specific molecules. Future work can apply these insights to continue understanding the repertoire of tastant-receptor interactions behind basic, canonical tastes and emerging, non-canonical taste modalities.

## Author contributions

CA: Writing – review & editing, Writing – original draft, Visualization, Conceptualization. JG: Writing – review & editing, Writing – original draft, Visualization, Conceptualization. KA: Writing – review & editing, Visualization. MS: Writing – review & editing, Writing – original draft, Visualization, Supervision, Funding acquisition, Conceptualization.
